# Differential PI(4,5)P_2_ sensitivities of TRPC4, C5 homomeric and TRPC1/4, C1/5 heteromeric channels

**DOI:** 10.1038/s41598-018-38443-0

**Published:** 2019-02-12

**Authors:** Juyeon Ko, Jongyun Myeong, Young-Cheul Shin, Insuk So

**Affiliations:** 1 Department of Physiology, Seoul National University College of Medicine, Seoul, 03080 Republic of Korea; 2 Department of Physiology and Biophysics, University of Washington School of Medicine, Seattle, WA 98195 USA; 3 Department of Cell Biology, Harvard Medical School, Boston, MA 02115 USA

## Abstract

Transient receptor potential canonical (TRPC) 4 and TRPC5 channels are modulated by the Gα_q_-PLC pathway. Since phosphatidylinositol 4,5-bisphosphate (PI(4,5)P_2_) maintains TRPC4 and TRPC5 channel function, the Gα_q_-PLC pathway inhibits channel activity by depleting PI(4,5)P_2_. Here we investigated the difference in PI(4,5)P_2_ sensitivity between homomeric and heteromeric TRPC channels. First, by using a *Danio rerio* voltage-sensing phosphatase (DrVSP), we show that PI(4,5)P_2_ dephosphorylation robustly inhibits TRPC4α, TRPC4β, and TRPC5 homotetramer currents and also TRPC1/4α, TRPC1/4β, and TRPC1/5 heterotetramer currents. Secondly, sensitivity of channels to PI(4,5)P_2_ dephosphorylation was suggested through the usage of FRET in combination with patch clamping. The sensitivity increased in the sequence TRPC4β < TRPC4α < TRPC5 in homotetramers, whereas when forming heterotetramers with TRPC1, the sensitivity was approximately equal between the channels. Thirdly, we determined putative PI(4,5)P_2_ binding sites based on a TRPC4 prediction model. By neutralization of basic residues, we identified putative PI(4,5)P_2_ binding sites because the mutations reduced FRET to a PI(4,5)P_2_ sensor and reduced the current amplitude. Therefore, one functional TRPC4 has 8 pockets with the two main binding regions; K419, K664/R511, K518, H630. We conclude that TRPC1 channel function as a regulator in setting PI(4,5)P_2_ affinity for TRPC4 and TRPC5 that changes PI(4,5)P_2_ sensitivity.

## Introduction

This article concerns the TRPC subfamily of ion channels, which includes seven gene members. TRPC channel subunits have six transmembrane domains with a pore loop between the 5^th^ and 6^th^ transmembrane domain. Four TRPC subunits combine to form functional tetrameric ion channels. TRPC4 and TRPC5 subunits can form homomeric channels, and they also form heteromeric channels with TRPC1^1–3^. Homo- and heteromeric TRPC channels are activated by stimulation of G-protein coupled receptors (GPCR) that induce hydrolysis of PI(4,5)P_2_^4,5^ and calcium release^6,7^.

The TRPC1 subunit may be devoted to a regulatory role in heteromeric channels rather than forming homomeric functional channels. Changes in the biophysical properties by the heteromerization support that formation of TRPC channel complexes may account for the importance of the role of the TRPC1 channel^8,9^. *In vivo* data proposed that combination of TRPC1, C4 and C5 forms a functional nonselective cation channels in brain^8,10^. Besides, defect in regulation of TRPC1 is related to several diseases such as diabetic nephropathy, Parkinson’s disease and Huntington’s disease. However, the specific functional role and mechanism of TRPC1 channel remain unclear.

Phosphoinositides (PIs) are essential membrane lipids that regulate a wide variety of cellular functions including membrane trafficking, cytoskeleton dynamics, cell migration, cytokinesis, and fluxes of ions and metabolites across the membrane^11–13^. PI(4,5)P_2_ is the PI signaling molecule that is located primarily in the plasma membrane inner leaflet. Its effects are complex, and research on the actions of PI(4,5)P_2_ on channels is ongoing. Many ion channels, including calcium channels^4,5,14,15^ and potassium channels^16–19^ are known to be regulated by PI(4,5)P_2_. GPCR signaling coupled to Gα_q_ activates PLC and in turn hydrolyzes PI(4,5)P_2_ into inositol triphosphate (IP_3_) and diacylglycerol (DAG). Recently, many methods to regulate the intracellular PI(4,5)P_2_ have been developed to test how PI(4,5)P_2_ affects channel activities. They include a rapamycin-inducible system^16^, a light-dependent optogenetic system^3^, and the depolarization mediated voltage-sensitive phosphatase (VSP) system^20^. These methods offer opportunities to explore poorly understood functional roles of PI(4,5)P_2_ on TRPC4 and TRPC5 channels^7,15,21^.

In a precedent study in our group, Kim *et al*. used chemical-control system to manipulate PI(4,5)P_2_ content in the plasma membrane^15^. This paper proposed that PI(4,5)P_2_ is essential for maintaining the activity of TRPC4β. Recently, also Myeong *et al*. reported activation of Gα_q_-coupled receptors leads to heteromeric TRPC1/4 and TRPC1/5 channels current inhibition^22^. Our previous works showed that the heteromeric channels are stimulated by direct interaction with activate Gα_q_, and subsequent PI(4,5)P_2_ hydrolysis makes channel to be inactivated. However, details in interaction between PI(4,5)P_2_ and channels are not well established.

In the present study, we set out to determine the difference in PI(4,5)P_2_ dependence of TRPC4, C5 homomers and heteromers with TRPC1 and to identify specific regions of possible lipid interaction. We verified that dephosphorylation of PI(4,5)P_2_ by different approaches efficiently inhibited TRPC4 and TRPC5. The different homomeric channels have different sensitivity to PI(4,5)P_2_, but the different heteromeric channels show identical PI(4,5)P_2_ sensitivity suggesting a dominant regulatory action of TRPC1 channels. Using a molecular model of the TRPC4 channel, we find several positively charged cytosolic residues of TRPC4 that seem to contribute to putative PI(4,5)P_2_ binding sites.

## Results

### PI(4,5)P_2_ depletion using lipid phosphatases

We began by studying the effects of PI(4,5)P_2_ depletion on TRPC4 and TRPC5 channel currents. The currents were recorded by patch clamp while plasma membrane PI(4,5)P_2_ was monitored by imaging. The cells co-expressed TRPC4 or TRPC5, muscarinic receptor 3 (M_3_R), and the YFP-tagged pleckstrin homology (PH) domain of PLCδ, a probe that binds PI(4,5)P_2_^23^. Two splice variants of TRPC4 were used: the long, full-length TRPC4α, and a short version TRPC4β that lacks 84 amino acids (785–868) in the C-terminus^24–26^. Stimulating the Gα_q_-coupled M_3_R with carbachol (CCh, 100 μM) evoked transient activation of current followed by concurrent robust depletion of PI(4,5)P_2_ (see Supplementary Fig. S1). We suspect that the decay of TRPC4 and TRPC5 channel currents is a consequence of the depletion of PI(4,5)P_2_.

The Gα_q_-PLC pathway not only depletes PI(4,5)P_2_ but also generates several other potent signals such as DAG, PKC, and Ca^2+^. To isolate the pure effect of PI(4,5)P_2_ depletion on channel currents, we turned to activation of three lipid phosphatases. We started with a rapamycin-inducible dimerization system that translocates an inositol polyphosphate 5-phosphatase (Inp54p) enzyme to the plasma membrane^16,27^. HEK293 cells were co-transfected with channel subunits, Lyn-FRB and CFP-FKBP-Inp54P together. Channel currents were first activated by a new agonist for TRPC4 and TRPC5 channels, Englerin A (EA, 100 nM)^28,29^. In cells co-expressing CFP-FKBP-Inp54p and Lyn-FRB, application of rapamycin inhibited channel currents gradually (see Supplementary Fig. S2). As a second test of lipid phosphatases, we used an optogenetic system that depletes PI(4,5)P_2_ by light^3^. Channels are co-expressed with CIBN fused to a C-terminal CAAX sequence (CIBN-CAAX) and CRY2 fused to the OCRL 5′-phosphatase (CRY2-OCRL). After 1 minute of EA, blue-light illumination (425–440 nm) substantially reduced channel currents amplitude in cells transfected with CRY2-OCRL (see Supplementary Fig. S3).

**Figure 1:**
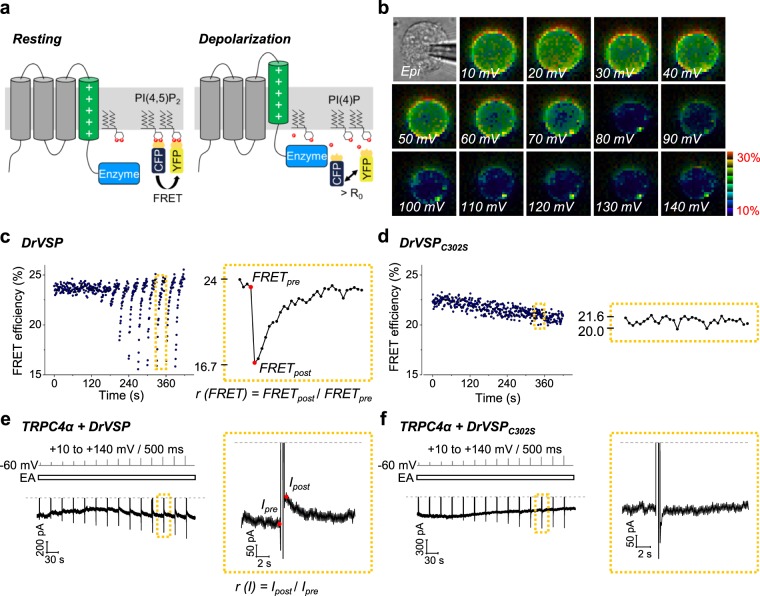
DrVSP mediated PH probe FRET reduction and current inhibition. (**a**) Principles of measurement of PI(4,5)P_2_ sensor FRET in cells transfected with PI(4,5)P_2_ sensor (PLCδ1 PH-domain) fused to CFP or YFP and DrVSP. (**b**) Pseudocolor FRET images of PI(4,5)P_2_ sensor during progressive depolarization from +10 to +140 mV in 10 mV steps. (**c**,**d**) FRET efficiency change caused by step-pulse protocol (from +10 to +140 mV; duration of 500 ms; repeated every 30 s) from cells transfected with CFP-PH, YFP-PH and DrVSP (**c**) or DrVSP_C302S_ (**d**). The area enclosed by the dashed box includes enlarged form (*right*). (**e**,**f**) Current change caused by step-pulse protocol from cells transfected with TRPC4α and DrVSP (**e**) or DrVSP_C302S_ (**f**). Englerin A (EA, 100 nM) was applied to induce the currents. The area enclosed by the dashed box includes enlarged form (*right*).

As a final approach to depleting PI(4,5)P_2_, we used the voltage-sensitive lipid phosphatase DrVSP, a lipid phosphatase that converts PI(4,5)P_2_ to PI(4)P upon plasma membrane depolarization. The changes of PI(4,5)P_2_ were monitored in real time using a co-transfected FRET pair consisting of donor (CFP) and acceptor (YFP) each fused to PH domain of PLCδ (Fig. 1a)^23^. DrVSP was activated by a strong depolarizing voltage pulse applied to the cell in whole-cell recording mode. Initially, most of the fluorescent PI(4,5)P_2_ indicators lay at the plasma membrane and reported high FRET efficiency, but then during depolarizing steps, they dispersed into the cytoplasm and the mean FRET efficiency dropped (Fig. 1b). A 500-ms step-pulse voltage was applied and incremented at 30 s intervals progressively from +10 to +140 mV. With large depolarizations, the FRET efficiency decayed quickly as PI(4,5)P_2_ was dephosphorylated, and it returned quickly between pulses (Fig. 1c). As a control, when a phosphatase loss-of-function mutant (DrVSP_C302S_)^20^ was used, there was no PI(4,5)P_2_ reduction (Fig. 1d). As expected, the loss of FRET efficiency was graded with the depolarizing pulse amplitude. Plotting the ratio FRET_post_/FRET_pre_ for each pulse (defined in Fig. 1c *right*) against voltage shows the PI(4,5)P_2_ falling progressively to a new steady state as the 500-ms voltage step was increased (see Supplementary Fig. S4). This decline was absent in cells expressing the non-functional DrVSP_C302S_ mutant.

We then measured channel currents in cells co expressing DrVSP and TRPC4α. The TRPC4α current induced by 100 nM EA was robustly inhibited after each depolarizing pulse (the ratio I_post_/I_pre_ is defined in Fig. 1e) in parallel with the FRET reduction. The phosphatase activity of the VSP also showed time dependency. As the duration prolonged, it showed gradational current decrease (see Supplementary Fig. S5). On the other hand, there was no current inhibition in cells expressing the DrVSP_C302S_ mutant (Fig. 1f). Collectively, the results of activating M_3_R, FKBP-Inp54p, CRY2-OCRL, and DrVSP support the hypothesis that PI(4,5)P_2_ is essential for TRPC4 and TRPC5 channel activity so that channel activity falls whenever PI(4,5)P_2_ is depleted.

### Inhibition of current in TRPC4, C5 homotetramers and TRPC1/4, C1/5 heterotetramers by DrVSP

**Figure 2:**
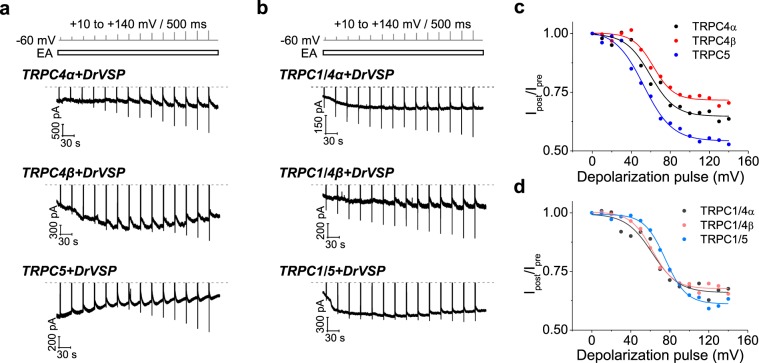
DrVSP-mediated current inhibition of TRPC4, TRPC5 homotetramers and TRPC1/4, TRPC1/5 heterotetramers. (**a**,**b**) Gradual current inhibition in TRPC4α, TRPC4β and TRPC5 homotetramers (**a**) and TRPC1/4α, TRPC1/4β and TRPC1/5 heterotetramers (**b**). The conditions are largely same as in Fig. 1e except transfected channels. (**c**,**d**) Current inhibition for different voltages in homotetramers (**c**) and heterotetramers (**d**).

We compared the effect of PI(4,5)P_2_ reduction on homomeric and heteromeric TRPC4α, TRPC4β and TRPC5 channel currents using the VSP-activating step-pulse protocol. All channel forms tested were sensitive to PI(4,5)P_2_ reduction (Fig. 2a,b). The inhibition occurred in cells expressing DrVSP and not in cells expressing DrVSP_C302S_ (see Supplementary Fig. S6). Plotting the current inhibition against the pulse voltage reveals small differences among the channel types. Among the homotetramers, TRPC5 showed relatively the most current inhibition after a weak DrVSP activation, with pulses ranging from +10 to +50 mV that hardly affected TRPC4α or TRPC4β (Fig. 2c). This result suggests that TRPC5 has a lower apparent affinity for PI(4,5)P_2_ than the other homotetramers. The EA-induced current in TRPC4β channels exhibited the least current inhibition from the depolarizing pulses even with the largest depolarizations. Correspondingly, the voltage of half maximal inhibition (V_half_), differed among homotetramers: TRPC4α (59 ± 3 mV), TRPC4β (62 ± 2 mV), TRPC5 (53 ± 2 mV). In contrast, the V_half_ of the three heteromeric channels is shifted to more positive voltages: TRPC1/4α (62 ± 3 mV), TRPC1/4β (63 ± 2 mV) and TRPC1/5 (75 ± 1 mV) (Fig. 2d). In its regulatory role, the TRPC1 channel raises the apparent PI(4,5)P_2_ affinity of heterotetrameric channels while retaining dependence on PI(4,5)P_2_.

### Quantification of homo- and heteromeric TRPC1/4/5 channel activity affected by PI(4,5)P_2_ depletion

**Figure 3:**
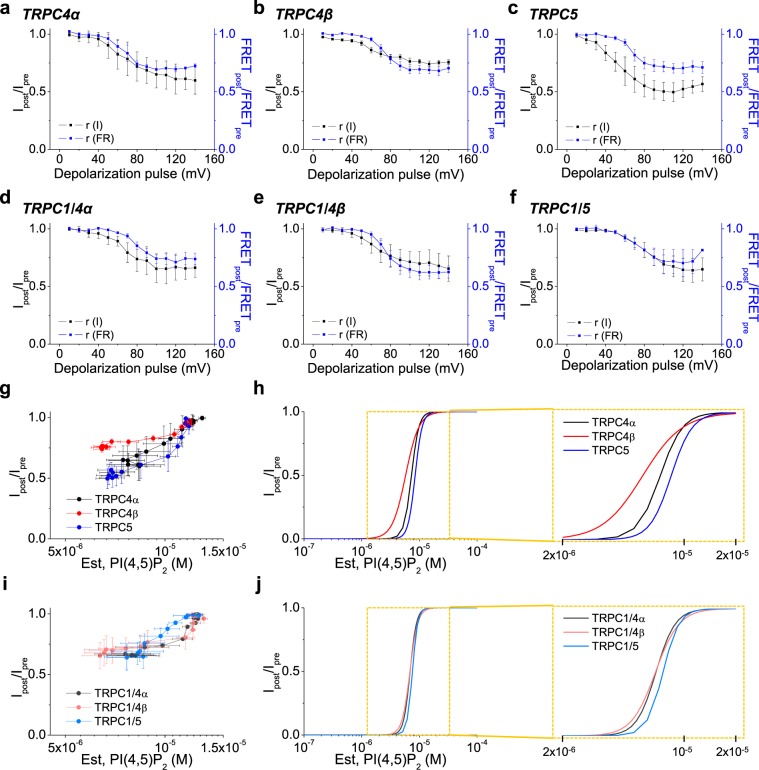
Quantification of PI(4,5)P_2_ dissociation from binding to homo- and heteromeric channels. (**a**–**f**) Ratio of voltage dependent current inhibition and FRET reduction after DrVSP activation in cells expressing TRPC4α (n = 6) (**a**), TRPC4β (n = 7) (**b**) and TRPC5 (n = 10) (**c**) homotetramers and TRPC1/4α (n = 4) (**d**), TRPC1/4β (n = 8) (**e**) and TRPC1/5 (n = 7) (**f**) heterotetramers. (**g**) *r(I)* against estimated PI(4,5)P_2_ concentration plots based on the conversion from FRET to PI(4,5)P_2_ of homotetramers. (**h**) Hill plots for homotetramers, enclosed by the dashed box is at a higher PI(4,5)P_2_ concentration resolution. (**i**,**j**) The conditions are largely same as in Fig. 3g,h, except heterotetramers instead.

To quantify the differential sensitivities of homomeric TRPC4, TRPC5 and heteromeric TRPC1/4, TRPC1/5 channels better, the currents were recorded by patch clamp while plasma membrane PI(4,5)P_2_ was monitored by FRET imaging. The normalized channel current inhibition *r(I)* and FRET reduction *r(FR)* were plotted against the voltage of the depolarizing pulses used to activate DrVSP. The cells co-expressed channel subunits (with no fluorescent tag), DrVSP, and CFP/YFP fused PH-domains. Looking first at the three homotetramers, their current inhibition roughly paralleled the FRET reduction, but the depth of current inhibition was greatest for TRPC5 and least for TRPC4β (Fig. 3a–c). Thus, the sensitivity to a large PI(4,5)P_2_ depletion increases in order TRPC4β < TRPC4α < TRPC5. As before, in the same experiment done with heterotetramers, the curves were more similar (Fig. 3d–f).

The FRET between CFP-PH and YFP-PH as they bind to PI(4,5)P_2_ at the plasma membrane is a non-linear indicator of the PI(4,5)P_2_ surface density. The PI(4,5)P_2_ concentration was estimated from changes in FRET using a previously suggested square-law equation^5^.

1 $$\frac{FR}{F{R}_{max}}\approx {F}^{2}={(1/(1+\frac{{K}_{d(PHd)}}{[PI(4,5){P}_{2}]}))}^{2}$$

Here K_*d(PHd)*_ is the dissociation constant of the complex of PH domains bound to PI(4,5)P_2_, suggested to be ~2.0 μM^30,31^. FR_max_ is the FRET ratio at an infinite concentration of PI(4,5)P_2_, which we estimated by artificially increasing the PI(4,5)P_2_ level using PIP5K to generate more PI(4,5)P_2_. Overexpression of PIP5K raised the FRET efficiency to 1.5 times that in control cells (control: 19.8 ± 1.9%, PIP5K: 30.7 ± 1.9%) (see Supplementary Fig. S7). Normalized channel current inhibition *r(I)* was plotted against the PI(4,5)P_2_ concentration estimated from the FRET using Eq. 1 (Fig. 3g,i). The data values were fitted using the function to produce a sigmoid curve and to determine the effective concentration (EC_50_) of each TRPC channels for a comparison (Fig. 3h,j). This gave estimated functional PI(4,5)P_2_ dissociation constants for the homotetrameric TRPC4β, TRPC4α, and TRPC5 channels of 6, 7, and 9 μM, respectively (Fig. 3h, *right box*). Estimated functional PI(4,5)P_2_ dissociation constants for heterotetramers were in the same range: 7, 7 and 8 μM (Fig. 3j, *right box*). Again, looking at the fitted curves, the homomeric channels differ more than heterotetramers suggesting that TRPC1 subunits tend to normalize PI(4,5)P_2_ sensitivities.

### Putative sites of PI(4,5)P_2_ regulation of TRPC4

**Figure 4:**
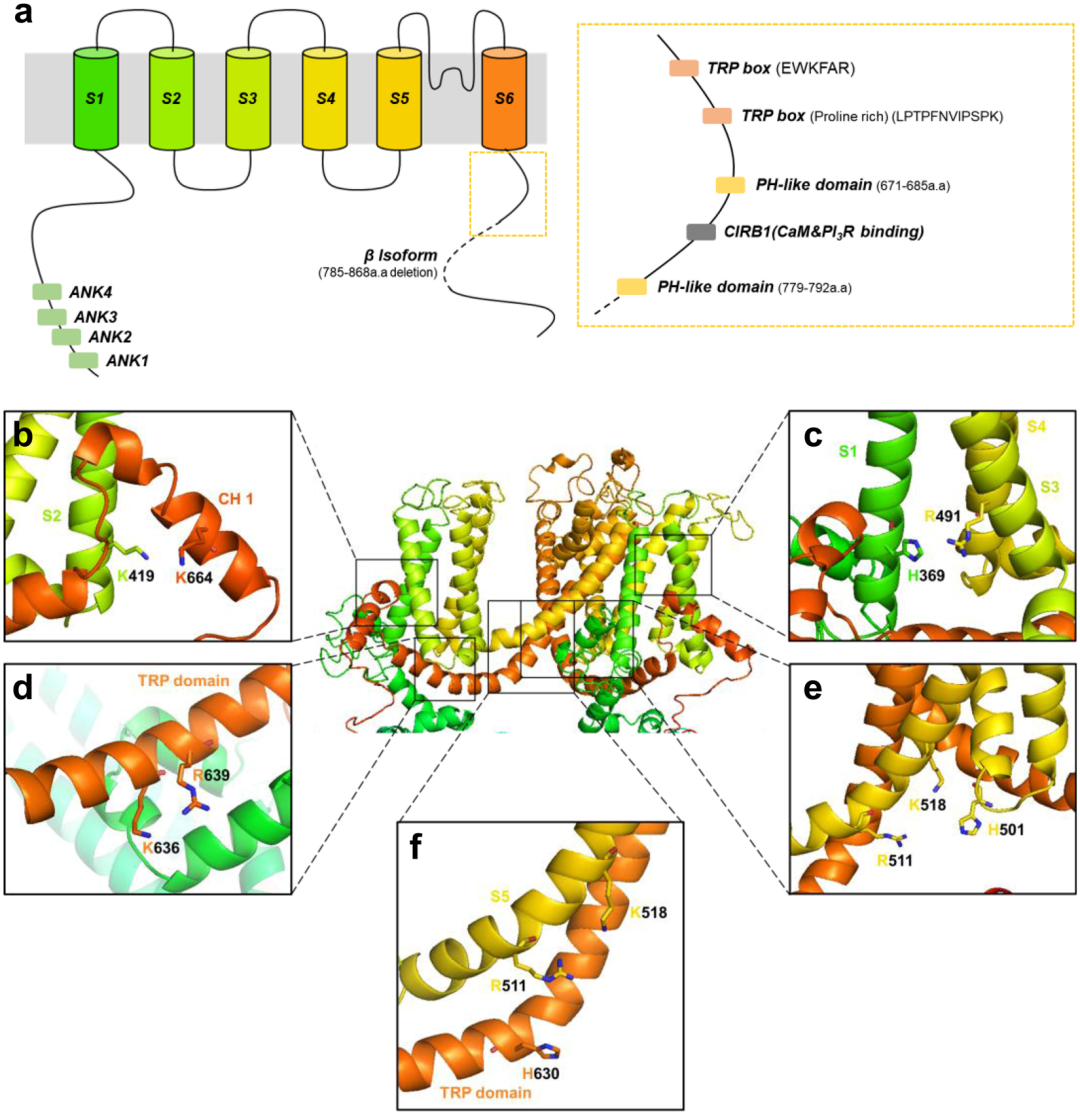
Molecular model of TRPC4 based on the structure of NOMPC and TRPM4. (**a**) Schematization of a TRPC4 subunit. (**b**–**f**) Expended views of putative PI(4,5)P_2_ binding regions in prediction model of TRPC4.

TRPC channel subunits have 6 transmembrane domains and cytosolic N- and C- termini; they are expressed on the plasma membrane in a punctate distribution (Fig. 4a). The C-terminus includes two PH-like domains and two TRP boxes of which one, EWKFAR, is conserved in other TRP family members, and the other is proline rich. To identify potential amino acids and structural motifs for PI(4,5)P_2_ binding in the TRPC4 channel, we generated a molecular model of the channel using template-based model prediction based on known TRPM4 and NOMPC channel structures. We hypothesized that some positively charged amino acids near the cytoplasmic face of the channel should be in electrostatic contact with PI(4,5)P_2_ in the plasma membrane. Accordingly, potential binding pockets for PI(4,5)P_2_ in the template-based model are shown in enlarged form (Fig. 4b–f). A lipid could bind tightly to exposed basic residues on the channel, such as K419 and K664 (Fig. 4b), H369 and R491 (Fig. 4c) or K636 and R639 (Fig. 4d) of one subunit. Contrastingly, the hydrophobic cleft formed by R511 and K518 of one subunit and H501 of its adjacent subunit (Fig. 4e) is also a candidate for PI(4,5)P_2_ interaction. K518 corresponds to a site suggested to be an interaction site in TRPV1^32^ and TRPM8^33^ channel. Also, H630 instead of H501 in one subunit is a candidate for PI(4,5)P_2_ interaction (Fig. 4f).

**Figure 5:**
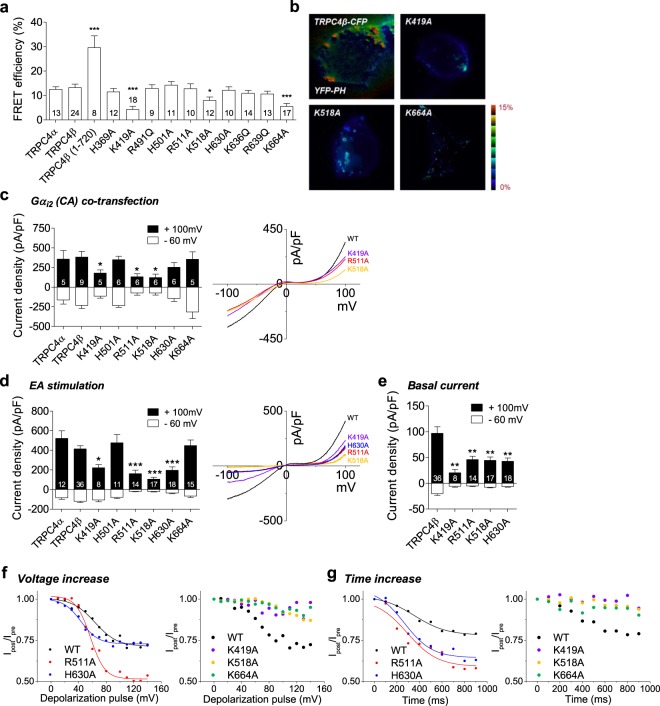
Putative PI(4,5)P_2_ binding sites of TRPC4 and its functional change. (**a**) Summary of FRET efficiency of TRPC4 mutants. (**b**) Images of FRET between CFP tagged channels (wild-type TRPC4β and mutants K419, K518A and K664A) and YFP-PH. (**c**) Current potentiation of wild-type TRPC4 or mutant channels transfected with Gα_i2_ (Q209L). The I-V relationships of wild-type and mutants which shows significantly reduced currents (*right*). (**d**) EA (100 nM) stimulation of wild-type TRPC4 or mutant. The I-V relationships of wild-type and mutants (*right*). (**e**) Basal current density of mutants which showed decreased activities comparison with wild-type TRPC4β. (**f**,**g**) Current inhibition for different voltages (**f**) or different time durations (**g**) in WT, R511A, H630A (*left*) and K419A, K518A, K664A (*right*). Data are presented as mean ± SEM and analyzed using student’s t-test. *P < 0.05, **P < 0.01, ***P < 0.001.

Several of the candidate basic residues were tested by neutralization. Interaction between channel and PI(4,5)P_2_ was detected by FRET between the CFP tagged TRPC4 channel mutants and YFP tagged PH-domain which expressed in HEK293 cells (Fig. 5a,b). It is assumed that the PH-domain was attracted and gathered around the channel, due to the channel is binding with PI(4,5)P_2_. In other words, TRPC4 channel, PI(4,5)P_2_, and PH-domain do not bind all together at the same time, but PI(4,5)P_2_ can bind to TRPC4 or PH-domain competitively. Thus, wild-type channel and PH-domain shows FRET mediated by PI(4,5)P_2_. Alanine substitution of K419 in the cytoplasmic domain in the S2 helix, K518 in S5, and K664 in after TRP domain of C-terminus result in significantly reduced FRET efficiency. A deletion mutant of TRPC4β (1–720) showed high FRET compare to WT which suggests that a short C-terminus makes interaction between CFP and YFP closer. The other tested mutants showed no FRET differences. These results suggest that TRPC4 and PI(4,5)P_2_ have several interaction sites including K419, K518 and K664 and that together these sites may contribute to regulation by PI(4,5)P_2_. Hence, pocket1 (K419A and K664) and pocket2 (H501, R511 and K518) or pocket3 (R511, K518 and H630) which includes low FRET mutants are good candidates for a PI(4,5)P_2_ binding region.

### Functional tests of putative PI(4,5)P_2_ binding sites of TRPC4

The mechanism by which PI(4,5)P_2_ interaction sites regulate TRPC4 channels is not known. To investigate functional changes in channel activity, channel currents of wild-type and mutant TRPC4 were recorded with ramp voltage protocol from −100 mV to +100 mV in whole cell recording. For a more physiological stimulation, we co-expressed channels with the constitutively active form of Gα_i2_ (Q209L) in HEK293 cells, which is known to activate TRPC4 channels^34^ (Fig. 5c). As our measure of channel activity, we took the difference between enhanced currents in Cs^+^-rich external solution and currents in normal Na^+^-rich solution. Figure 5c plots the difference current densities at +100 mV and −60 mV. The current was significantly smaller for three of the basic residue mutations: K419A, R 511A, and K518A. It was no significant change for mutations H501A, H630A, or K664A. No major alterations in the I-V curve were observed in these mutants with double rectifying shape (Fig. 5c *right*).

We also investigated the other channel activation pathway by stimulating channels with EA for further confirmation (Fig. 5d). Channels were stimulated by 100 nM EA for 2 min, and channel currents were recorded with the same ramp pulse protocol as in Gα_i2_ (Q209L) experiments. EA stimulation corroborated the previous results: With EA, H501A, K664A, and wild-type TRPC4β current densities showed large enhancement. K419A, R511A, K518A and H630A showed less enhancement with characteristic I-V feature as a pore forming functional channel (Fig. 5d *right*). With these latter mutants, the basal currents before EA stimulation were less than for wild-type TRPC4β. The K419A mutant channel displayed strongly reduced basal currents (Fig. 5e). In other TRPC families, these five sites are well conserved. In addition, these mutants are well targeted to the plasma membrane and form puncta as the wild-type channel (see Supplementary Fig. S8). Hence, the observed current density decrease with mutants could be due to disrupted electrostatic interaction of PI(4,5)P_2_ with the positively charged binding residues.

As a final approach to identify the evidence of an altered PI(4,5)P_2_ sensitivity in channel mutants, we compared the effect of PI(4,5)P_2_ reduction on mutant channels. The experiment was performed using the VSP-activating step-pulse protocol with identical process as shown in Fig. 2. The progressive current decrease was shown with lower depolarizations than wild-type for two of mutations: R511A (57 ± 2 mV) and H630 (43 ± 2 mV). The decline was almost absent in cells expressing K419A, K518A, K664A (Fig. 5f). We also examined using time-dependent VSP-activating protocol. Duration prolongation corroborated the previous results: With R511A (291 ± 46 ms) and H630A (267 ± 54 ms) current decreased significantly in shorter time than wild-type (355 ± 33 ms), and current change was diminished in K419A, K518A and K664A (Fig. 5g). Hence, these results demonstrate that R511A, H630A has a lower apparent affinity for PI(4,5)P_2_ than the wild-type TRPC4. In addition, K419A, K518A and K664A completely lost dependence on PI(4,5)P_2_ by disturbance in channel-PI(4,5)P_2_ interaction.

Collectively, our findings suggest that K419, R511, K518, H630 and K664 are good candidates for PI(4,5)P_2_ binding sites. Therefore, in the TRPC4 channel, the putative PI(4,5)P_2_ binding region has two parts, a hydrophobic inner cleft and an outer surface region. Consequently, one functional tetrameric channel could bind 8 molecules of PI(4,5)P_2_.

## Discussion

In the present study, we demonstrate that: 1) The TRPC1 subunit regulates the PI(4,5)P_2_ interaction of channels containing TRPC4 or TRPC5 subunits. 2) The sensitivity to PI(4,5)P_2_ is different for each channel in the sequence TRPC4β < TRPC4α < TRPC5. Heteromeric channels are regulated to have more similar sensitivities by inclusion of TRPC1 subunits. 3) TRPC4 and PI(4,5)P_2_ may have several interactions in two binding regions, so that more than one molecule of PI(4,5)P_2_ may bind to each subunit. The several TRPC tetramers are initially activated when transfected M_3_ muscarinic receptors are stimulated^22^. Further study is needed to understand the mechanism of this channel activation.

The TRPC4 channel is known to be regulated by PI(4,5)P_2_, however the molecular details of this regulation have remained elusive. Storch *et al*.^21^ suggested that PI(4,5)P_2_ depletion increases 1-Oleoyl-2-acetyl-glycerol (OAG)-induced currents in TRPC5 expressing cells. However, in our hands, application of EA (100 nM), after wortmannin (Wtn, 20 μM), phosphoinositide PI-4 kinase inhibitor, showed decreased channel activity than control (see Supplementary Fig. S9). Our study aimed to determine the function of PI(4,5)P_2_ itself on channels, in which the degradation of PI(4,5)P_2_ is involved in channel inactivation. On the other hand, Storch *et al*.^21^ and Thakur *et al*.^7^ have suggested PI(4,5)P_2_ as an activation accelerator in DAG or Gα_i_-mediated activation. Therefore, these studies suggest a possibility of different effects caused by an influence of PI(4,5)P_2_ with other molecules, and further experiments need to be studied.

Here, we provide direct evidence that TRPC4, C5 homomeric- and TRPC1/4, TRPC1/5 heteromeric channels are maintained by PI(4,5)P_2_, so that PI(4,5)P_2_ dephosphorylation results in current inhibition. In TRPC4, the PI(4)P that is generated by PI(4,5)P_2_ dephosphorylation in our three systems did not participate in maintaining function, unlike for TRPV1 which can be maintained by PI(4)P and other phosphoinositides as well as by PI(4,5)P_2_^35^. There are numerous different approaches for the study of phosphoinositide signaling, such as rapamycin-inducible system, light-dependent system and depolarization mediated VSP system. We mainly used the VSP system, which allowed quantitative analysis and did not require addition of chemicals.

Our, quantitative analysis using VSP showed that homomeric TRPC4 and TRPC5 channels have different sensitivities to PI(4,5)P_2_ in the order TRPC4β, TRPC4α and TRPC5. This difference in the homomeric channels presumably reflects affinity differences for PI(4,5)P_2_. In a preceding study of TRPC3, 6, 7 channels, the functional dissociation constants for PI(4,5)P_2_ binding to channels were estimated at 1, 2 and 5 μM, respectively^5^. In comparison, TRPC4, 5 gave relatively higher values.

The calcium permeability of TRPC4 and TRPC5 channels is changed when TRPC1 subunits are co-expressed with them^9^. We also have recently proposed that heteromerization of TRPC4 with TRPC1 decreased the calcium rise as detected by channels tagged with GCaMP6s^36^. Here, our data showed that estimated functional PI(4,5)P_2_ dissociation curves of TRPC1/4, TRPC1/5 heteromeric channels are not very different, which means that introducing TRPC1 subunits normalizes their PI(4,5)P_2_ sensitivities. We conclude that the inserted TRPC1 subunits in TRPC1/4 and TRPC1/5 heterotetramers modify many properties of the channels. The mechanism by which TRPC1 subunits affect the other TRPC channels remains unclear.

The sites of interaction of PI(4,5)P_2_ with other TRP subfamilies have been investigated. It has been proposed that basic residues in the TRP box are important in TRPM6^37^, TRPM8^33^ and TRPV1^32^ channels. Additionally, other positively charged cytosolic residues have been suggested as putative PI(4,5)P_2_ binding regions^38^. Following studies in other TRP channels, we thought to find the PI(4,5)P_2_-TRPC4 interaction sites. The published structure of the TRPV1 channel shows that PI(4,5)P_2_ binding site prediction based on the structure is quite successful. We have presented here a molecular model that used published structures of TRPV1, NOMPC and TRPM4 channels as a template. After this analysis was completed, a structure for TRPC4 was published^39,40^. The transmembrane domain of the new cryo-electron microscopy structure is remarkably like our molecular model (see Supplementary Fig. S10). Then, after S6 comes the TRP domain, two proline-rich segments, and the C-terminus. We placed K664, which is a residue comes after TRP domain, near the edge of the inner leaflet. As in the structures of TRPC3 and TRPC4, the C-terminal region was considered highly flexible and was not be resolved. Also our model could not include the full C-terminal region. Even the second proline-rich region including residues P661 and P663 was not resolved in the cryo-EM structure so we cannot make a comparison to our structure prediction for K664.

We used FRET method to identify the directly interacting residues. The PI(4,5)P_2_ binding is a competitive electrostatic interaction with PH-domain and positively charged residues of the channel. Therefore, putative binding site mutants cannot attract the PI(4,5)P_2_ probe as a wild-type channel, since the electrostatic contact between the mutant and PI(4,5)P_2_ is disturbed. These results also support that the interaction between channel and PI(4,5)P_2_ can be identified by FRET. We have obtained the enhanced FRET signal with TRPC4 (1–720) but similar FRET values in isoforms. We cannot clarify how TRPCα and β exactly form differently at plasma membrane, because the structure of 84a.a (785–868 a.a) region, that different in isoforms, is not identified^39^. However, according to Otsuguro *et al*.^41^, this region was suggested to be attached to the plasma membrane, hence we expect that difference in distance from the membrane to fluorophore does not affect FRET signal significantly. In the case of 1–720, the distance to the membrane is reduced because the lower part is completely deleted (254 a.a missing). Therefore, a short C-terminus makes the distance of CFP and YFP closer.

We mutated basic residues predicted by the model as potential PI(4,5)P_2_ binding sites in TRPC4. They fell in two spatial clusters: the first with K419 and K664 and the second with R511, K518 and H630. We expected mutations of basic residues to decrease the apparent affinity for PI(4,5)P_2_, and the activity of the channels. FRET with the PI(4,5)P_2_ indicator was reduced in K419A, K518A and K664A mutants. In electrophysiological characterization, two of these mutants K419A and K518A (but not K664A) also showed decreased channel activity both with constitutive activation by Gα_i2_ (Q209L) and with exogenous stimulation by EA. The experiment using the VSP-activating voltage or time increase step-pulse protocol showed that R511A and H630A responded fast for PI(4,5)P_2_ reduction, and current change was totally diminished in K419A, K518A and K664A. Therefore, it is likely that the basic residues, K419 and K518 participate in direct channel-PI(4,5)P_2_ interaction. K664 also interacts closely with PI(4,5)P_2_ (FRET, VSP); however, it does not play a decisive role in channel activity. Other mutants, R511A and H630A, showed a current decrease and PI(4,5)P_2_ affinity alteration without a FRET change. Presumably they do not interact directly with PI(4,5)P_2_, but may support the K518 site to tightly bind with PI(4,5)P_2_. Therefore, we envision two PI(4,5)P_2_ binding regions in each TRPC4 subunit one near the cytoplasmic surface region and the other in a hydrophobic inner cleft.

Collectively, our data reveal that PI(4,5)P_2_ is required for maintenance of all homomeric TRPC4, TRPC5 and heteromeric TRPC1/4, TRPC1/5 channels. Each channel form has a different PI(4,5)P_2_ sensitivity, and the TRPC1 subunit modulates the sensitivities. Further experiments are required in heteromeric channels to understand the origin of this effect of TRPC1 subunits. We identified key domains of TRPC4 for lipid binding, identifying 2 pockets that might bind PI(4,5)P_2_. If there are two binding regions, there might be several states of occupancy depending on PI(4,5)P_2_ affinities for each pocket, and the functional homomeric TRPC4 channel may bind up to eight PI(4,5)P_2_ molecules.

## Materials and Methods

### Cell culture and transfection

Human embryonic kidney (HEK293) cells were incubated in Dulbecco’s Modified Eagle’s Medium (DMEM) supplemented with 10% heat-inactivated FBS and penicillin (100 units/ml), streptomycin (100 μg/ml) at 37 °C in 5% CO_2_ humidified incubator. All mutants were generated by Quickchange mutagenesis (Agilent Technologies), using the sense and antisense primers. Every plasmid DNA was prepared using a plasmid midi kit (QIAGEN), according to the manufacturer’s instructions. All constructs were confirmed by DNA sequencing. Cells were seeded in 12-well plate for whole-cell patch clamp and imaging. The following day, transfection was carried out by using the FuGENE^®^ 6 Transfection Reagent (Promega). All experiments were performed 20–30 h after transfection.

### Solutions and drugs

The patch pipette containing standard intracellular solution; 140 mM CsCl, 10 mM HEPES, 0.5 mM EGTA, 3 mM Mg-ATP, 0.2 mM Tris-GTP, pH 7.3 with CsOH. In the reproducibility experiment, only the EGTA concentration was changed to 0.05 mM. External solution was perfused constantly as follows; 5 mM KCl, 10 mM HEPES, 1 mM MgCl_2_, 135 mM NaCl, 2 mM CaCl_2_, 10 mM glucose, pH 7.4 with NaOH. The Cs^+^-rich external solution contained equimolar CsCl rather than NaCl and KCl. (−)Englerin A (EA) was purchased from phytoLab.

### Electrophysiological recordings

The cells were transferred onto a small chamber on the stage of an inverted microscope (IX70, OLYMPUS, Japan) and attached to coverslip in the small chamber for 10 min prior for the patch recording. Glass microelectrodes with 2–2.5 megaohms resistance were used to obtain gigaohm seals. The currents were recorded using an Axopatch 200B patch-clamp amplifier (Axon instrument, USA). The whole cell configuration was used to measure the TRPC4 channel current in the HEK293 cells. Voltage ramps ranging from +100 to −100 mV over period of 500 ms were imposed every 10 sec with a holding membrane potential of −60 mV. pCLAMP software (version 10.2, Axon Instruments, USA) were used for data acquisition and the data were analyzed using the OriginPro 8 (OriginLab, USA) and GraphPad Prism 5 (GraphPad Software Inc., USA).

### Imaging and FRET measurements

To obtain the image of a cell, we used an inverted microscope (IX70, OLYMPUS, Japan) with 60X oil objective lens. Each image was captured on an EMCCD camera (iXon3, ANDOR, Northern Ireland) under the control of MetaMorph7.6 software (Molecular devices, USA) system.

### FRET efficiency computation

FRET measurements were made by the three-cube FRET method^42^. The FRET Ratio (FR) is equal to the fractional increase in YFP emission due to FRET and was calculated as *FR* = *F*_*AD*_ *AD/FA* = *[S*_*FRET*_*(DA)*−*R*_*D1*_ *· S*_*CFP*_*(DA)]/(R*_*A1*_ *· [S*_*YFP*_*(DA)−R*_*D2*_ *· S*_*CFP*_*(DA)])*. Here, S_CUBE_(SPECIMEN) denotes an intensity measurement, where CUBE indicates the filter cube (CFP, YFP, or FRET), and SPECIMEN indicates whether the cell is expressing the donor (D; CFP), acceptor (A; YFP), or both (DA). R_D1_ = S_FRET_(D)/S_CFP_(D), R_D2_ = S_YFP_(D)/S_CFP_(D), and R_A1_ = S_FRET_(A)/S_YFP_(A) are predetermined constants from measurements applied to single cells expressing only CFP- or YFP-tagged molecules. Although three-cube FRET does not require that CFP and YFP fusion constructs preserve the spectral features of the unattached fluorophores, similar ratios and recorded spectra furnished two indications that the spectral features of the fluorophores were largely unperturbed by fusion. Since the FR relies on YFP emission, YFP should be attached to the presumed limiting moiety in a given interaction. Subsequent quantitative calculations based on FR relied on a presumed 1:1 interaction stoichiometry. The effective FRET efficiency (E_EFF_) was determined by E_EFF_ = E · A_b_ = (FR−1) · [E_YFP_(440)/E_CFP_(440)], where E is the intrinsic FRET efficiency when fluorophore-tagged molecules are associated with each other, Ab is the fraction of YFP-tagged molecules that are associated with CFP-tagged molecules, and the bracketed term is the ratio of YFP and CFP molar extinction coefficients scaled for the FRET cube excitation filter^43^. We determined this ratio to be 0.094 based on maximal extinction coefficients for ECFP and EYFP^44^ and excitation spectra.

### Statistical analysis

All statistical analysis was done with GraphPad Prism 5. Results were compared using two-way ANOVA and Student’s t-test. A probability value (*P*) that less than 0.05 was considered statistically significant. Data are presented as means ± SEM; *P < 0.05, **P < 0.01, ***P < 0.001.

### Homology modeling of the transmembrane region in TRPC4

The predicted three-dimensional (3D) structure of TRPC4 was achieved by combination of template-based homology modeling and secondary structure prediction. First, we submitted the full sequence of TRPC4 and simply obtained homology models from multiple PDB depositions by the employment of the I-TASSER^45^. Since only TM4-TRP domain of TRPC4 was matched on TRPV1, NOMPC and TRPM4^46–48^, we utilized a secondary structure prediction server^49^ (JPred4) for characterizing the N-terminus region before TM4. Based on secondary structure, highly possible models were carefully selected that fulfilled our criteria for use as an initial prediction model. We obtained the final model of TRPC4 monomer, which was generated from NOMPC (N-terminal cytosol domain) and TRPM4 (transmembrane domain) by specify template with alignment. The TRPC4 monomer model was duplicated to four chains and superimposed to the TRPM4 tetramer (PDB code: 6BCO) and merged for assembling a functional tetrameric complex. After tetramer assembly, energy minimization was run for reducing clashed residues between chain-chain interaction phase, by using of UCSF Chimera^50^. Target-template superimposition, merging and the schematic representations were performed by Pymol 1.7.4.4 (Schrodinger, LLC; San Diego, CA).

## Supplementary information

Supplementary figures
