# Supplementary material for: Differential PI(4,5)P2 sensitivities of TRPC4, C5 homomeric and TRPC1/4, C1/5 heteromeric channels
Source: Sci Rep. 2019 Feb 12;9:1849. doi: 10.1038/s41598-018-38443-0 (PMC6372716; doi:10.1038/s41598-018-38443-0)
Supplement: Supplementary file 1 — Supplementary figures [file 41598_2018_38443_MOESM1_ESM.pdf]

Supplementary information

Differential PI(4,5)P_2 sensitivities of TRPC4, C5 homomeric and TRPC1/4, C1/5 heteromeric channels

Juyeon Ko [a], Jongyun Myeong [a,b], Youngcheul Shin [c], Insuk So [a*]

[a] Department of Physiology, Seoul National University College of Medicine, Seoul, 03080, Republic of Korea, [b] Department of Physiology and Biophysics, University of Washington School of Medicine, Seattle, WA 98195, U.S.A, [c] Department of Cell Biology, Harvard Medical School, Boston, MA 02115, U.S.A

Juyeon Ko and Jongyun Myeong contributed equally to this work.

*Corresponding author:

Insuk So, Department of Physiology, Seoul National University College of Medicine, Seoul, 110-799, Republic of Korea. E-mail address: insuk@snu.ac.kr, Tel: (82) 2-740-8228, Fax: (001) 82-2-763-9667

Supplementary Figures and Figure legends

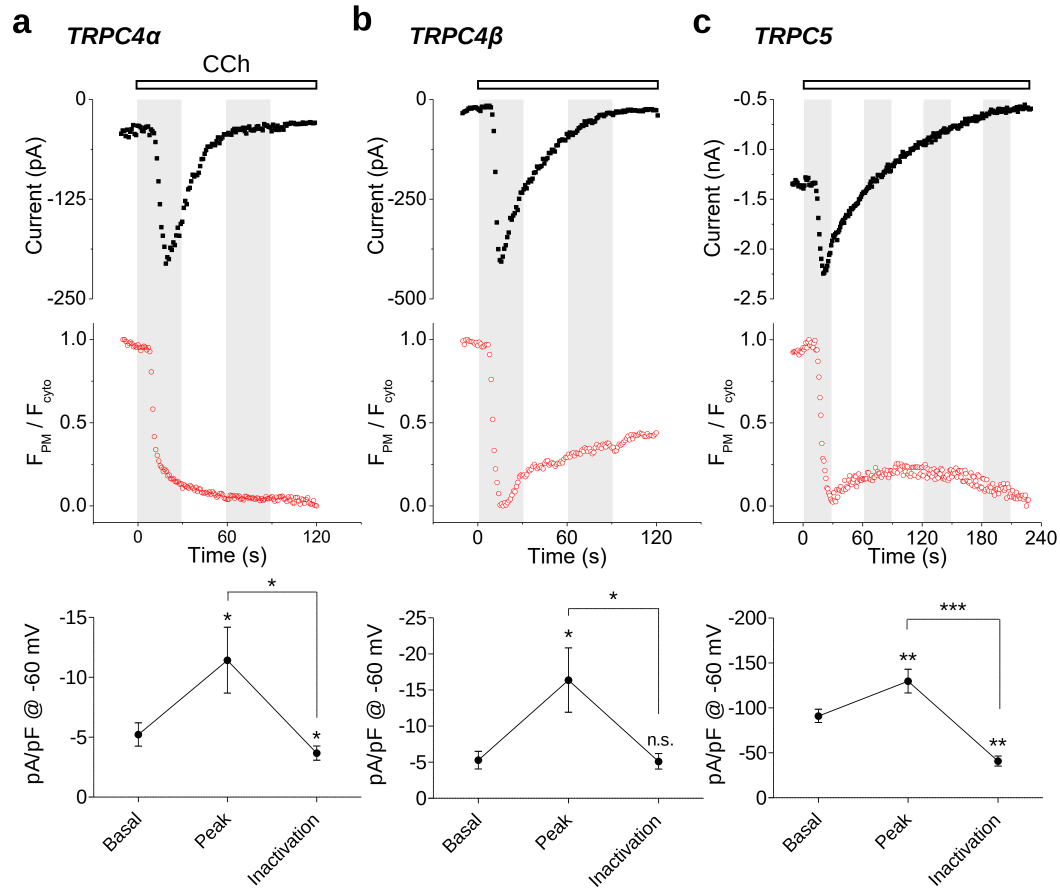

Supplementary Figure 1. **The inhibited TRPC4α, TRPC4β and TRPC5 currents by the PI(4,5)P_2 depletion**

(a) Trace of TRPC4α currents (*top*), fluorescence intensity ratio of plasma membrane and cytosol (F_{PM}/F_{cyto}) of PI(4,5)P_2 sensor (*middle*) and summary of basal, peak and inactivation current densities (*bottom*) upon 100 μM CCh stimulation to activate M_3R. **(b, c)** Same as in **a**, but in cells expressing TRPC4β **(b)** or TRPC5 **(c)**.

Data are presented as mean ± SEM and analyzed using student's t-test. *P < 0.05, **P < 0.01.

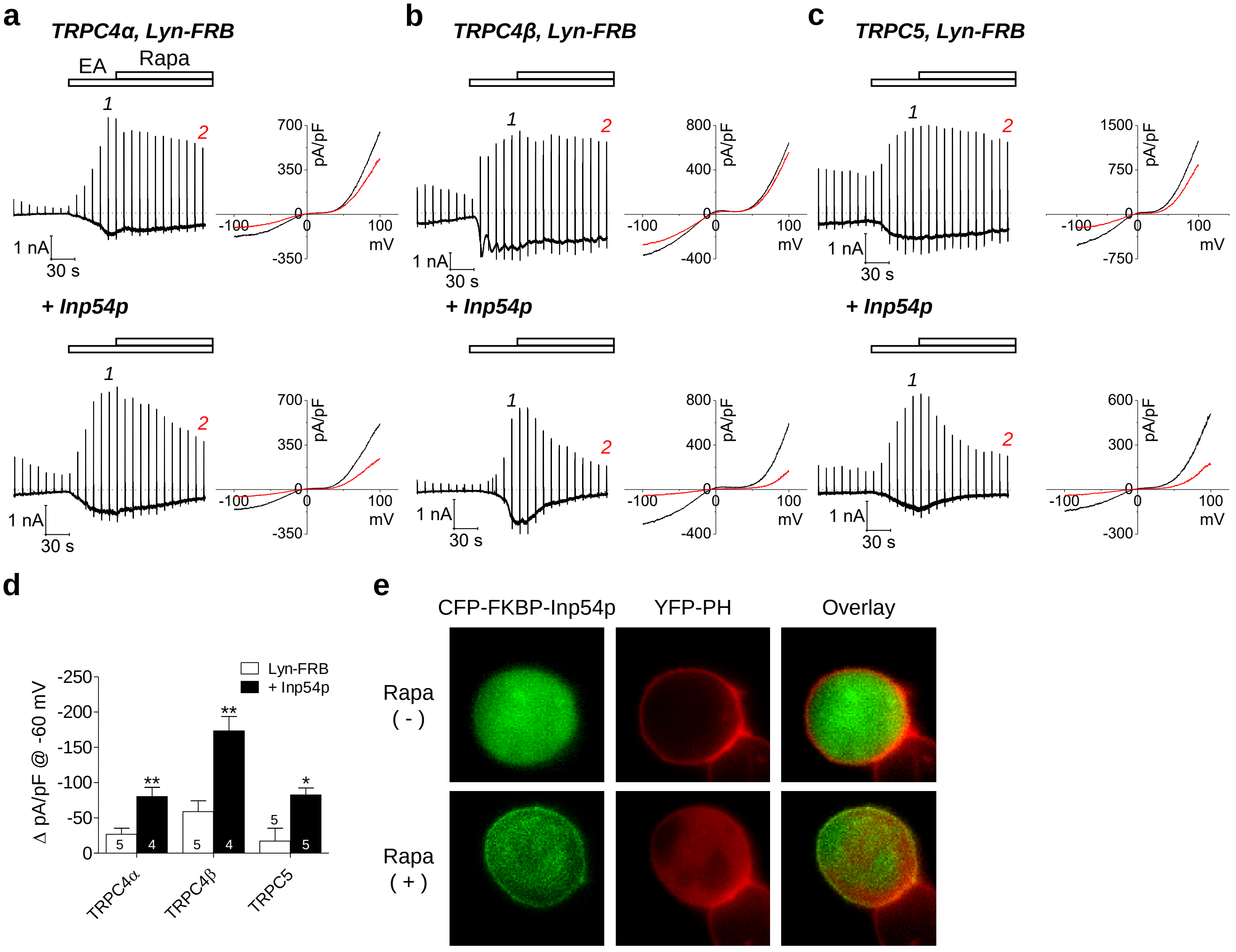

Supplementary Figure 2. **Pure effect of PI(4,5)P_2 depletion on channel currents by rapamycin inducible system**

(a-c) Rapamycin (Rapa, 20 nM) induced current change in TRPC4α **(a)**, TRPC4β **(b)** and TRPC5 **(c)** after EA (100 nM) stimulation. Whole-cell current and I-V curve in the cells transfected with Lyn-FRB alone (*upper panel*) or Inp54p together (*bottom panel*). A plasma-membrane-targeted FRB domain of mTOR (Lyn-FRB) can be heterodimerized with the 5'-phosphatase tagged FKBP by adding rapamycin. Application of rapamycin recruits the Inp54p phosphatase to the plasma membrane anchor Lyn-FRB, converting PI(4,5)P_2 to PI(4)P by hydrolyzing the 5' phosphate. **(d)** Summary of *Δ* current density at -60 mV between EA evoked peak and subsequent addition of rapamycin. **(e)** Translocation of CFP-FKBP-Inp54p and YFP-PH by 20 nM rapamycin (Rapa). A 20 nM concentration of rapamycin was sufficient to recruit CFP-FKBP-Inp54 to the membrane and to induce PI(4,5)P_2 depletion

Data are presented as mean ± SEM and analyzed using student's t-test. *P < 0.05, **P < 0.01.

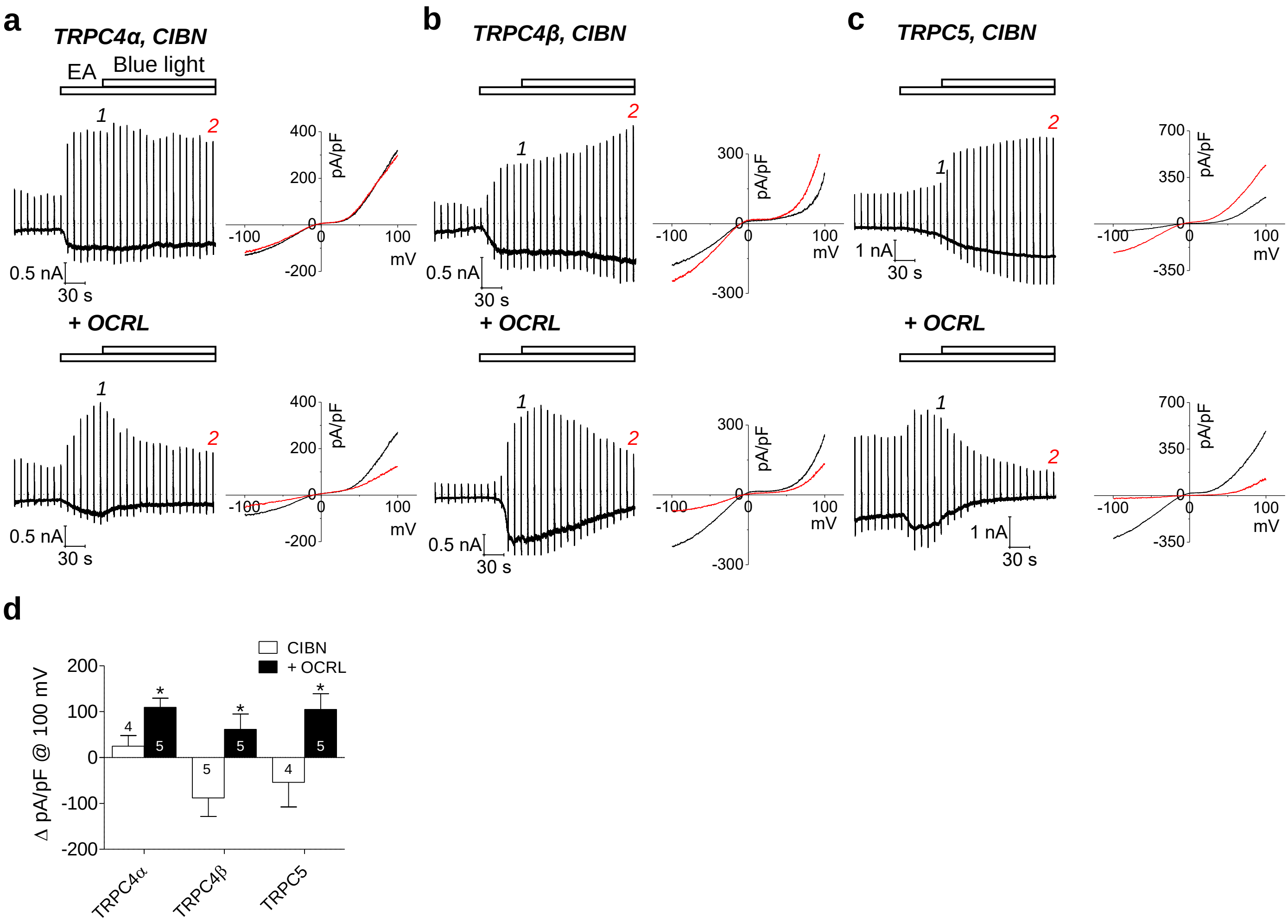

Supplementary Figure 3. **Pure effect of PI(4,5)P_2 depletion on channel currents by optogenetic system**

(a-c) Blue-light induced current inactivation in TRPC4α **(a)**, TRPC4β **(b)** and TRPC5 **(c)** after 100 nM EA stimulation. Whole-cell current and I-V curve in the cells transfected with CIBN alone (*upper panel*) or OCRL together (*bottom panel*). Blue-light induces dimerization of CRY2 fused to the OCRL 5'-phosphatase (CRY2-OCRL) to the plasma-membrane targeted CIBN fused to a C-terminal CAAX sequence (CIBN-CAAX) which recruits a 5'-phosphatase domain to the plasma membrane. **(d)** Summary of *Δ* current density at + 100 mV between EA evoked peak and subsequent blue-light illumination.

Data are presented as mean ± SEM and analyzed using student's t-test. *P < 0.05.

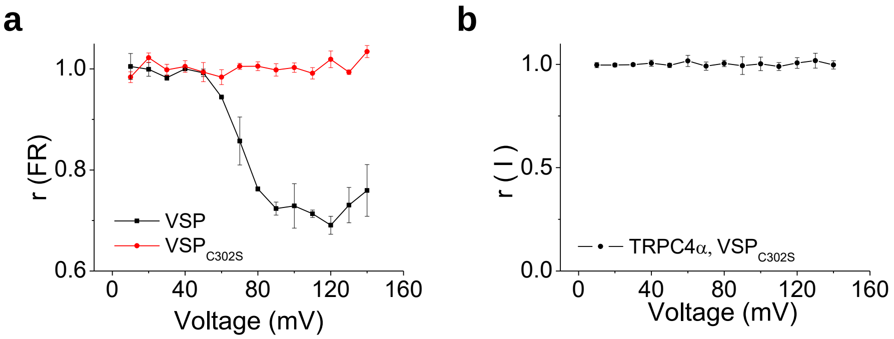

Supplementary Figure 4. **Failed PI(4,5)P_2 depletion by enzyme-defective mutant DrVSP$_{C302S}$**

(a) Normalized FRET of the PI(4,5)P_2 sensor expressed as the ratio $FRET_{post}/FRET_{pre}$ following depolarizing step pulses in the presence of DrVSP or DrVSP$_{C302S}$. **(b)** Normalized TRPC4α current expressed as the ratio I_{post}/I_{pre} following depolarizing step-pulses in the presence of DrVSP$_{C302S}$.

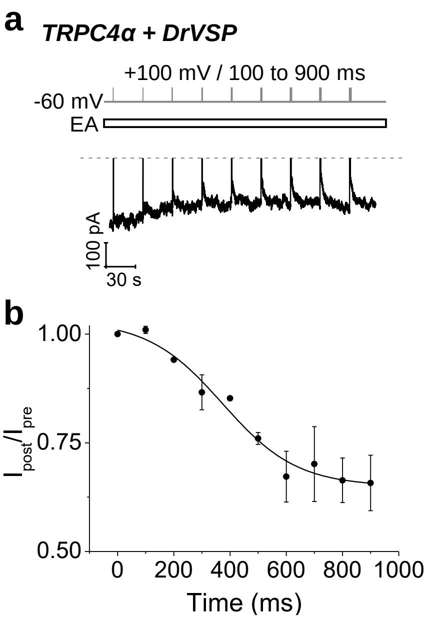

Supplementary Figure 5. **Time dependent phosphatase activity of DrVSP**

(a) Current change caused by step-pulse protocol (+100 mV; from 100 ms to 900 ms; repeated every 30 s) from cells transfected with DrVSP and TRPC4α **(b)**. Normalized TRPC4α current expressed as the ratio I_{post}/I_{pre} following depolarizing step pulses in the presence of DrVSP.

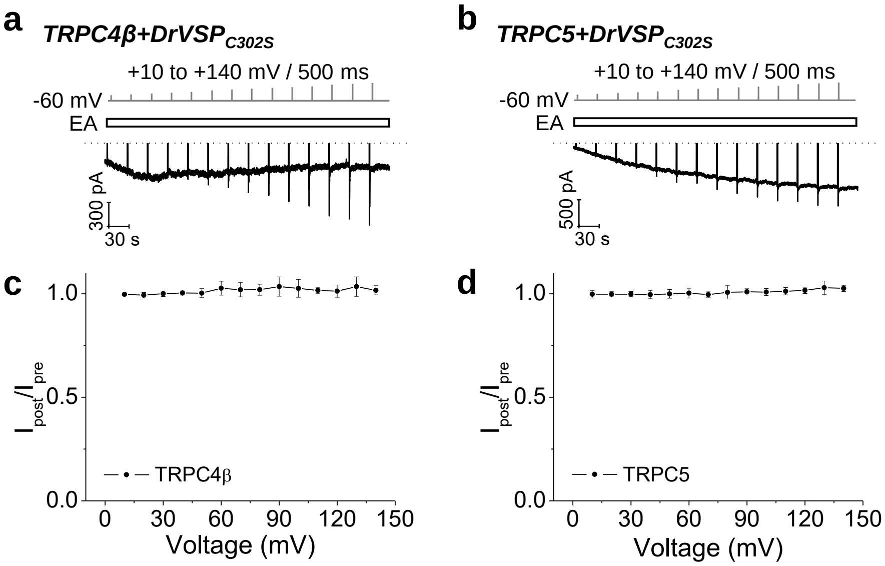

Supplementary Figure 6. **Failed current inhibition by enzyme-defective mutant DrVSP$_{C302S}$**

(a, b) Current change caused by step-pulse protocol (from +10 to +140 mV; duration of 500 ms; repeated every 30 s) from cells transfected with DrVSP$_{C302S}$ and TRPC4β **(a)** or TRPC5 **(b)**. **(c, d)** Current inhibition ratio of TRPC4β **(c)** and TRPC5 **(d)** elicited by depolarization step-pulse in the presence of DrVSP$_{C302S}$.

a

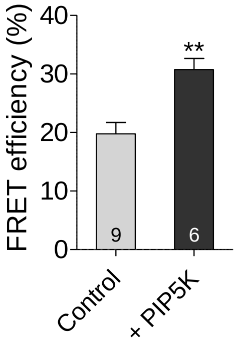

Supplementary Figure 7. **Artificially increased PI(4,5)P_2 level by overexpressing PIP5K**

(a) Comparison of FRET efficiency in the resting condition between cells expressing CFP-PH, YFP-PH (control), and additional PIP5K expression.

Data are presented as mean ± SEM and analyzed using student's t-test. **P < 0.01.

a

```
                  419                                 511      518
TRPC1   435  IWSDIKRLWYE  445   527  QISMGQMLQDFGKFLGMF  544
TRPC4   414  IWGEIKQMWDG  424   506  QISLGRMLLDILKFLFIY  523
TRPC5   415  IWGEIKEMWDG  425   507  QISLGRMLLDILKFLFIY  524
                  630                                 664
TRPC1   650  QLIANHEDKEW  660   684  IIPSPKTICYM  694
TRPC4   625  QLIADHADIEW  635   659  VIPSPKSLWYL  669
TRPC5   629  QLIADHADIEW  639   663  IIPSPKSFLYL  673
```

b

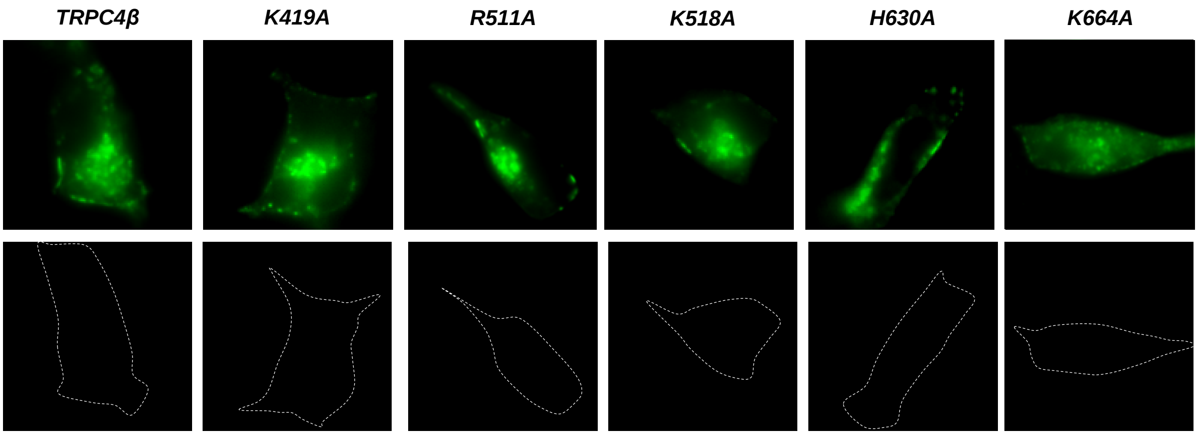

Supplementary Figure 8. **Conserved PI(4,5)P_2 binding basic residues**

(a) Amino acid sequence alignment of the TRPC4 putative PI(4,5)P_2 binding sites with other TRPCs. The conserved basic residues are highlighted red. **(b)** Images of wild-type TRPC4β and mutants. The mutants are well targeted to the plasma membrane.

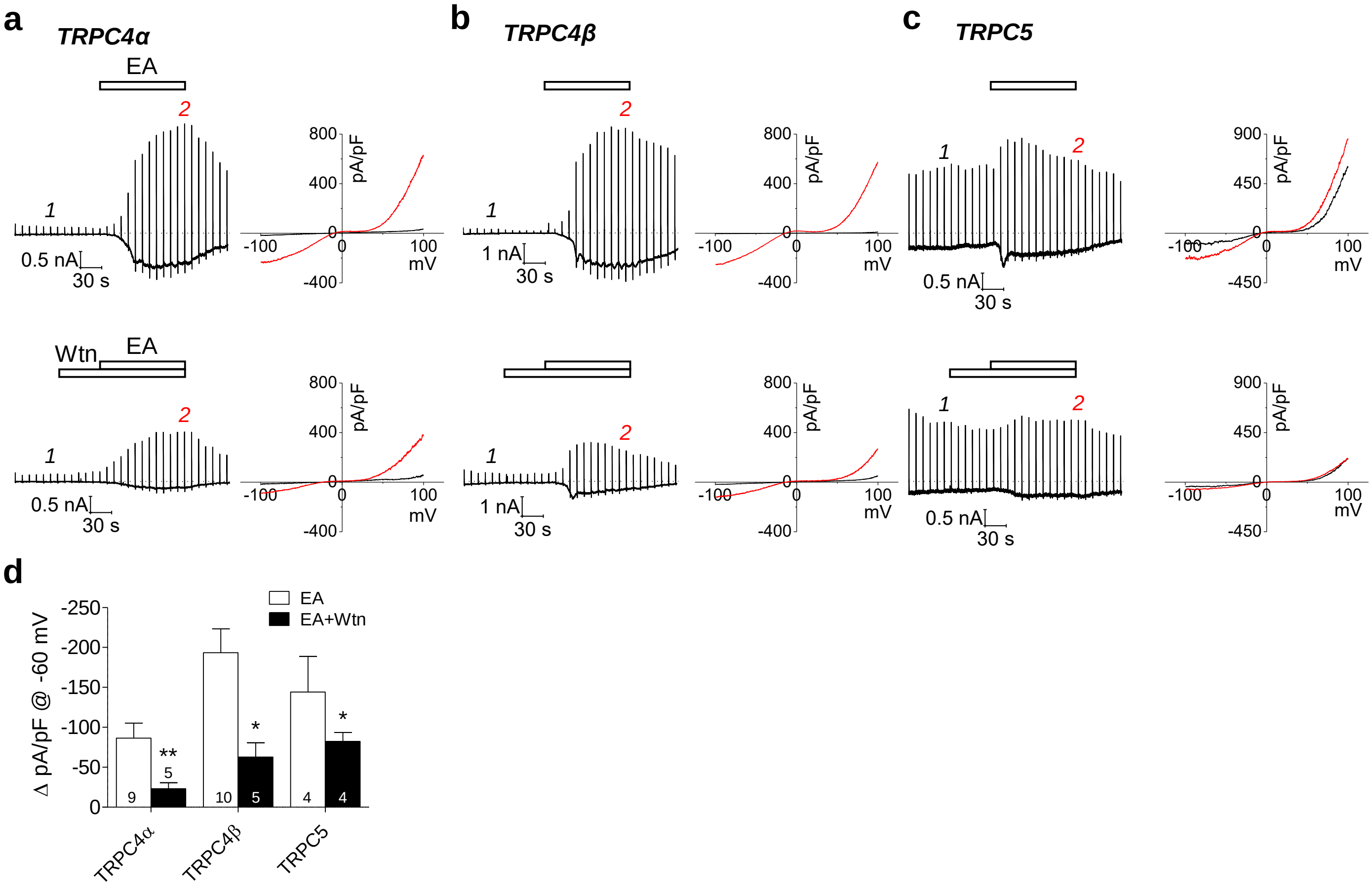

Supplementary Figure 9. **Pure effect of PI(4,5)P_2 depletion on channel currents by optogenetic system**

(a-c) EA induced currents in TRPC4α **(a)**, TRPC4β **(b)** and TRPC5 **(c)** after without (*upper panel*) or with (*bottome panel*) Wortmannin (Wtn, 20μM). Whole-cell current and I-V curve in the cells transfected with channel. In TRPC4 and TRPC5 transfected cells, Wtn was applied for 1 minute before EA stimulation. Wtn itself had no effect on TRPC4α, TRPC4β, and TRPC5 currents **(d)** Summary of *Δ* current density at - 60 mV between EA and EA+Wtn evoked peak.

Data are presented as mean ± SEM and analyzed using student's t-test. *P < 0.05, **P < 0.01.

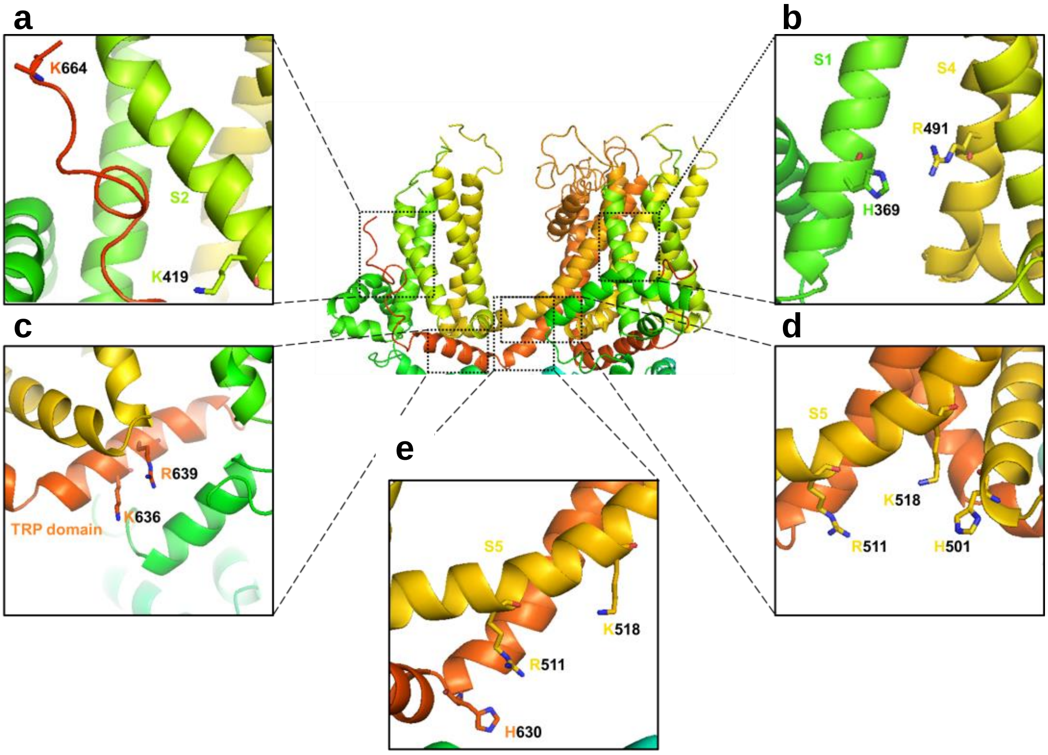

Supplementary Figure 10. **Putative PI(4,5)$_2$ binding regions in Cryo-EM structure of TRPC4**

(a-e) Expended views of putative PI(4,5)P$_2$ binding regions in Cryo-EM structure of TRPC4
